# Effects of Acidification and Preservatives on Microbial Growth during Storage of Orange Fleshed Sweet Potato Puree

**DOI:** 10.1155/2018/8410747

**Published:** 2018-06-07

**Authors:** Joyce Ndunge Musyoka, George Ooko Abong', Daniel Mahuga Mbogo, Richard Fuchs, Jan Low, Simon Heck, Tawanda Muzhingi

**Affiliations:** ^1^Department of Food Science, Nutrition and Technology, University of Nairobi, P.O. Box 29053-00625, Kangemi, Kenya; ^2^International Potato Center (CIP), Sub-Saharan Africa (SSA) Regional Office, Old Naivasha Road, P.O. Box 25171-00603, Nairobi, Kenya; ^3^Food and Markets Department, Natural Resources Institute of University of Greenwich, Central Avenue, Chatham Maritime, Chatham, Kent ME4 4TB, UK; ^4^International Potato Center (CIP), Regional Office, Plot 106, Katalima Road, Naguru, P.O. Box 22274, Kampala, Uganda

## Abstract

Orange Fleshed Sweet Potato (OFSP) puree, a versatile food ingredient, is highly perishable limiting its use in resource constrained environments. It is therefore important to develop shelf-stable puree. A challenge test study was carried out to determine the effect of combinations of chemical preservatives and acidification on microbial growth in stored puree. Puree was prepared and treated as follows: control (**A**); 0.05% potassium sorbate+0.05% sodium benzoate+1% citric acid (**B**); 0.1% potassium sorbate+0.1% sodium benzoate+1% citric acid (**C**); 0.2% potassium sorbate+0.2% sodium benzoate+1% citric acid (**D**); 1% citric acid (**E**). Samples were inoculated with* Escherichia coli* and* Staphylococcus aureus* at levels of 5.2 x 10^9^ cfu/100g and 1.5 x 10^9^ cfu/100g, respectively, before being evaluated during storage for 10 weeks at prevailing ambient temperature (15-25°C) and refrigeration temperature (4°C). Total aerobic counts, yeasts, and molds were also evaluated.* E. coli* and* S. aureus *counts declined significantly (p<0.05) by 4 log cycles in all puree treatments except for control and puree with only citric acid. Total viable count, yeasts, and molds were completely inhibited except for puree with only citric acid. Combination of chemical preservatives and acidification is effective in inhibiting pathogens and spoilage microorganisms in sweet potato puree.

## 1. Introduction

Sweet potato (*Ipomoea batatas*) is an important crop for food security and income generation in Sub-Saharan Africa (SSA) [1]. Sweet potato roots occur in various colors ranging from white, yellow, purple, and orange that is rich in *β*-carotene, an important pro-vitamin A carotenoid [2]. Biofortified orange fleshed sweet potato (OFSP) has been promoted in Kenya as an effective and sustainable source of vitamin A [3] that can be used to mitigate vitamin A deficiency (VAD), a major public health concern in the Western and Nyanza parts of Kenya [4].

In east Africa, OFSP roots are processed into puree (boiled and mashed) that is used at household level to make food products such as fried doughnuts (*mandazi*),* chapatti*, and porridges for children. OFSP puree is being used as a partial substitute for wheat flour in bakery products in Rwanda and Kenya. There are several economic advantages of using OFSP puree compared to OFSP flour. According to research, 1.25 kg of OFSP fresh roots makes 1 kg of puree while 4-5 kg of OFSP fresh roots is needed to make 1 kg of flour [5]. The major challenge in the use of OFSP puree is that it is highly perishable and requires refrigeration during storage and distribution. An advanced technology of processing sweet potato puree in the USA is done through sterilization and aseptic packaging using a continuous flow microwave system [6] giving a shelf-life of up to 36 months. However, this system is expensive in low income countries such as Kenya where OFSP puree is processed on small-scale.

Therefore, there is an urgent need for developing shelf-stable OFSP puree which does not require refrigeration to ensure a continuous supply for bakeries throughout the year. Shelf-stable OFSP puree will also help ensure all year supply of OFSP puree in countries with one sweet potato growing season. One of the approaches in producing shelf-stable OFSP puree is by controlling the growth of spoilage and pathogenic microorganisms through the use of ‘hurdles'. Hurdle technology is a method that ensures microbial stability and safety of foods as well as nutritional and sensory quality based on the application of several preservation factors [7]. The hurdles mainly used in food preservation include the use of preservatives, the type of packaging, temperature, water activity, and pH, among others [8].

Natural preservatives which give the much desired “clean label” have been shown to be effective against microbial growth in food products [9, 10]. However, natural preservatives such as nisin and natamycin are not cost-effective for small-scale processors in SSA. Chemical preservatives such as sodium benzoate (E211) and potassium sorbate (E202) are commonly used to retard or stop the growth of pathogenic microorganisms in food. Sodium benzoate and potassium sorbate are weak acids and their antimicrobial action is known to be due to the accumulation of protons and anions inside the microbial cell that disrupts normal metabolism [11]. Potassium sorbate and sodium benzoate are permitted in food products in levels of 0.1 % [12]. These two compounds have been used before in inhibiting microbial growth in food products. For instance, Jin [9] recorded the antimicrobial effect of sodium benzoate and potassium sorbate against* Escherichia coli* in strawberry puree. High acid foods such as fruit formulations require less heat treatment for their stability and therefore citric acid is added to maintain their pH at < 4.6 [13].

Citric acid (E330) also increases the acidity of the food product thus increasing the effectiveness of sodium benzoate and potassium sorbate since a greater proportion of the acids is in undissociated form [14]. Therefore, developing a shelf-stable OFSP puree using sodium benzoate, potassium sorbate, and citric acid may be effective as well as cost-effective. The quality and shelf-life of OFSP puree can be improved by application of alternative technologies for foodstuff packaging, distribution, and storage, such as modified atmosphere packaging (MAP) or vacuum packaging. MAP involves the replacement of the atmosphere surrounding food products with controlled mixtures of oxygen, nitrogen, and carbon dioxide before sealing in barrier materials [15]. During vacuum packaging the air is removed from within the packaging material and the product is enclosed with an airtight seal which prevents the return of air [16]. In SSA vacuum packing will be more cost-effective in the development of shelf-stable OFSP puree.

The potential growth of spoilage and pathogenic microorganisms in OFSP puree could affect its quality and shelf-life, as well as its safety. In order to determine the growth of pathogens in OFSP puree, a microbial challenge test (MCT) is important. This involves the inoculation of a food product with pathogenic microorganisms, storing the food under controlled conditions while evaluating the growth potential of the pathogens in the food product over time [17]. MCT is important as it helps in assessing whether a specific pathogen is able to grow in a food product if the properties of the food product such as pH are not able to control the particular pathogen [18]. MCT has been used to examine the growth potential of pathogens in foods in case of contamination. For instance, the growth and survival of pathogens such as* S. aureus* and* E. coli* in salad vegetables have been examined [17]. One important consideration in conducting a challenge test is selection of the appropriate pathogens. Contamination of OFSP puree with* S. aureus* could take place during postprocess handling of the puree by operatives [19]. This pathogen is commonly found on human skin and can be transferred to foods if good hygienic practices are not followed. OFSP puree could also be contaminated with* E. coli* through the use of nonpotable water for cleaning surfaces and equipment [20].* E. coli* has been known to survive and persist on surfaces for months [21]. These are real scenarios during OFSP puree processing at small-scale level in rural settings.

Therefore, the current study was designed to examine survival and growth potential of* S. aureus* and* E. coli* in stored OFSP puree with preservatives. Conducting the challenge test in OFSP puree will help in establishing the ability of potassium sorbate, sodium benzoate, and citric acid to inhibit the growth of* S. aureus*,* E.coli, *total counts, yeasts, and molds in OFSP puree held under ambient (15-25°C) and refrigeration (4°C) conditions and therefore facilitate more extensive use of the puree. Results from this study will determine the feasibility of developing shelf-stable OFSP puree which can be stored without refrigeration. Shelf-stable OFSP puree will bridge the OFSP puree shortage during off seasons and will also help reduce postharvest losses for OFSP farmers.

## 2. Materials and Methods

### 2.1. Design and Methodology

The current study employed an experimental study design by preparing orange fleshed sweet potato (OFSP) puree and carrying out microbial evaluation. The experimental study design included two independent variables: use of different combinations of preservatives and two different storage temperatures. The dependent variables were* E. coli* counts,* S. aureus* counts, total aerobic counts, and yeast and mold counts.

### 2.2. Preparation of OFSP Puree

**Figure 1:**
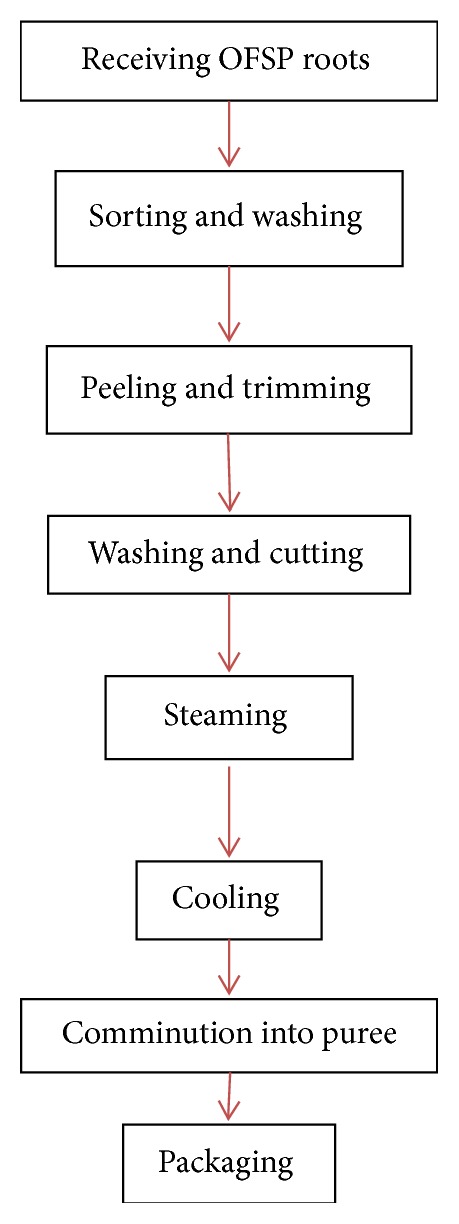
Preparation of OFSP puree.

About 25 kg of root samples was randomly collected from a pool of five batches from farmers in Homabay-Kenya. Kabode variety which is the most adopted and widely grown OFSP variety was used for the current study. The processed puree was packaged in 5-kilogram polyethylene bags of 300 micron and vacuum sealed before being transported overnight to the Department of Food Science, Nutrition and Technology (University of Nairobi) laboratory for evaluation.  Figure 1 shows a flow diagram of processing sweet potato puree. Sweet potato roots were sorted to remove the diseased and badly damaged roots before being washed to remove soil and loose dirt. The roots were then peeled manually using knives before being trimmed to remove the fibrous ends, surface blemishes, and the diseased ends of the roots. The washed roots were cut into approximately 0.5-0.75 cm slices in thickness and steamed at 100°C for 30 minutes in a steaming pot and then cooled for 30 minutes at ambient temperature. Cooked and cooled roots were then comminuted into puree using a pureeing machine (OMAS Food Machinery, AEE1T0, Euro ingredients limited, Italy), packaged in 5-kilogram polyethylene bags, and vacuum sealed using vacuum packaging machine (MINIPACK-TORRE S.p.A MVS 45X, ANNO-2015-Euro ingredients, Italy). This activity was carried out in the current puree processing plant in Kenya and with the help of workers in the plant in order to ensure that the study utilized similar product usually produced in the set up.

### 2.3. Bacterial Load in OFSP Puree

Before conducting the challenge test, OFSP puree was assessed microbiologically for* E. coli* and* S. aureus* and then microwaved and assessed again. The initial enumeration of* E. coli* and* S. aureus* in the puree was carried out as described in previous studies [22]. Puree sample (25 g) was homogenized with 0.85 % NaCl and serial dilutions were prepared up to 10^−6^. A volume of 0.1 mL from each dilution was spread in triplicate onto Brilliance* E. coli*/Coliform agar (Oxoid, Hampshire, England) and incubated at 37°C for 24 hours for enumeration of* E. coli*. Similarly, 0.1 mL of each dilution was spread in triplicate onto Baird parker agar (Oxoid, Hampshire, England) and incubated at 37°C for 48 hours for the enumeration of* S. aureus*. Enumeration was done for plates with 30-300 colonies. All microbial counts were expressed as mean base-10 logarithms of colony forming units per gram (log cfu/g). Data points were expressed as means from the triplicate analysis. The results indicated high levels of* E. coli *and* S. aureus* in puree before treatment with preservatives.* E. coli* and* S. aureus* were not detected after microwaving OFSP puree as shown in Table 1. This formed the basis for the level of inoculation of* E. coli* and* S. aureus* into the puree.

### 2.4. Preparation of Bacterial Inoculum

*Escherichia coli* ATCC 8739 and* Staphylococcus aureus* ATCC 6538 pellets were obtained from the American Type Culture Collection (Microbiologics, MN 56303-USA). The pellets were activated by suspending a single pellet of each microorganism in phosphate buffer (0.1 M) and incubating it at 38°C for 30 minutes. From the buffer, 1mL was transferred to nutrient broth and incubated at 35°C for 24 hours to allow for growth of the bacteria. The levels of inoculum obtained after plating were 5.2x10^9^ cfu/mL for* E. coli* and 1.5x10^9^ cfu/mL of* S. aureus*. The inoculum was then stored at -80°C to avoid changes that may affect growth [23].

### 2.5. Treatment of OFSP Puree Samples with Combination of Chemical Preservatives

OFSP puree was first sterilized for 3 minutes in a microwave. OFSP puree samples (100 g) were then dosed with combinations of selected chemical preservatives as shown in Table 2.

### 2.6. Inoculation Strategy and Growth Assessment

Puree samples (100 g) treated with preservative combinations and that without preservatives were inoculated with 1000 *μ*L of bacterial suspension containing 5.2 x 10^9^ cfu of* E. coli *and 1.5 x 10^9^ cfu of* S. aureus* resulting in a load of 5.2x10^7^ cfu (7.8 log cfu) of* E. coli*/g of puree and 1.5x10^7^ cfu (7.2 log cfu) of* S. aureus/*g of puree and vacuum sealed. Some inoculated samples were incubated at ambient (15-25°C) and others at refrigeration temperatures (4°C) with enumeration of bacterial load at weekly intervals. Serial dilutions of all samples were prepared up to 10^−6^ and each dilution was plated in triplicate for the different tested microorganisms. The experiments were performed independently three times.

### 2.7. Determination of Total Viable Count (TVC) and Yeast and Molds in OFSP Puree during Storage

OFSP puree was prepared as shown in Figure 1 with the addition of selected chemical preservatives (as in Table 2) before packaging 100 g in polyethylene bags. Some of the puree was packaged without preservatives and analyzed for TVC and yeasts and molds. The puree samples (100 g) were then stored at ambient temperature of (15-25°C) and refrigeration temperature (4°C) with TVC and yeast and mold evaluation weekly for a period of 10 weeks of puree storage. A sample of puree (25 g) was placed into 225 mL of sterile saline solution (0.85 % NaCl), vortexed for 1 minute to homogenize, and serially diluted to a dilution of 10^−7^. TVC was determined by transferring 1 mL of each sample dilution to sterile Petri dishes in triplicate to which approximately 20 mL of Plate Count Agar (PCA, LAB, UK) was added. The plates were swirled and allowed to solidify before being incubated at 30°C for 72 hours [24]. Yeasts and molds were determined by spread plating 0.1 mL of each sample dilution in triplicate onto Dichloran-Rose Bengal Chloramphenicol (DRBC) agar (Oxoid, Hampshire). The plates were incubated at 25°C for 5 days [25]. Enumeration was done for plates with 30-300 colonies. All microbial counts were expressed as mean base-10 logarithms of colony forming units per gram (log cfu/g). Data points were expressed as mean from the triplicate experiments and results were expressed as logarithm of colony forming units per gram (log cfu/g). The experiment was performed three times independently.

### 2.8. Determination of PH in OFSP Puree during Storage

One gram of OFSP puree sample was homogenized in 1mL of distilled water in a test tube. The pH values of the samples were measured using pH meter (model HI 98107, USA) by immersing the electrode directly into the sample in the test tube. Before the measurements, pH meter was calibrated using pH 4.0 and 7.0 buffers.

### 2.9. Statistical Analysis

Experiments were carried out in triplicate and quality control measures were taken into account. Data was analyzed by analysis of variance (ANOVA) using SPSS software (Version 20.0 SPSS Inc). Tukey test was used to determine the significant difference of mean values. The significance level was expressed at 5 % level. Microsoft Excel was used to plot line graphs.

## 3. Results

### 3.1. Changes in PH in OFSP Puree during Storage

**Figure 2:**
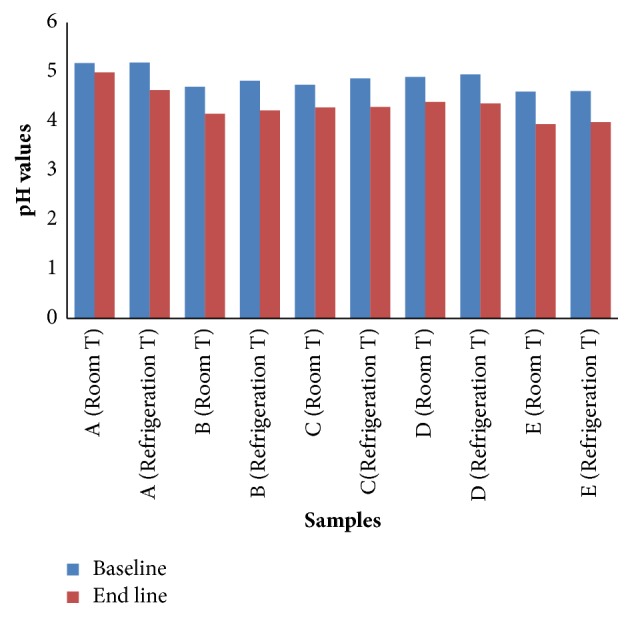
Changes in pH in OFSP puree during storage at ambient (15-25°C) and refrigeration temperature (4°C).** A**: puree without preservatives,** B**: puree with 0.05 % potassium sorbate+0.05 % sodium benzoate+1% citric acid,** C**: puree with 0.1 % potassium sorbate+0.1 % sodium benzoate+1% citric acid,** D**: puree with 0.2 % potassium sorbate+0.2 % sodium benzoate+1% citric acid, and** E**: puree with 1 % citric acid.

The initial pH of OFSP puree was 5.23 before treatment with preservatives. Addition of different combinations of sodium benzoate and potassium sorbate and citric acid led to a decline in pH of the puree kept at ambient (15-25°C) and refrigeration (4°C) conditions as shown in Figure 2.

Immediately after treatment of puree with preservatives, the highest pH values of 5.19 and 5.18 were obtained in the control sample at ambient and refrigeration temperatures, respectively, while least values of 4.60 and 4.61 were recorded in the sample with 1 % citric acid at ambient and refrigeration temperatures, respectively. At the end of the storage period (10 weeks), the highest pH values of 4.99 and 4.63 were recorded in the control sample, while least values of 3.94 and 3.98 were obtained in samples with 1 % citric acid at ambient and refrigeration temperatures, respectively.

### 3.2. Growth and Survival of* Escherichia coli* in Stored OFSP Puree

**Figure 3:**
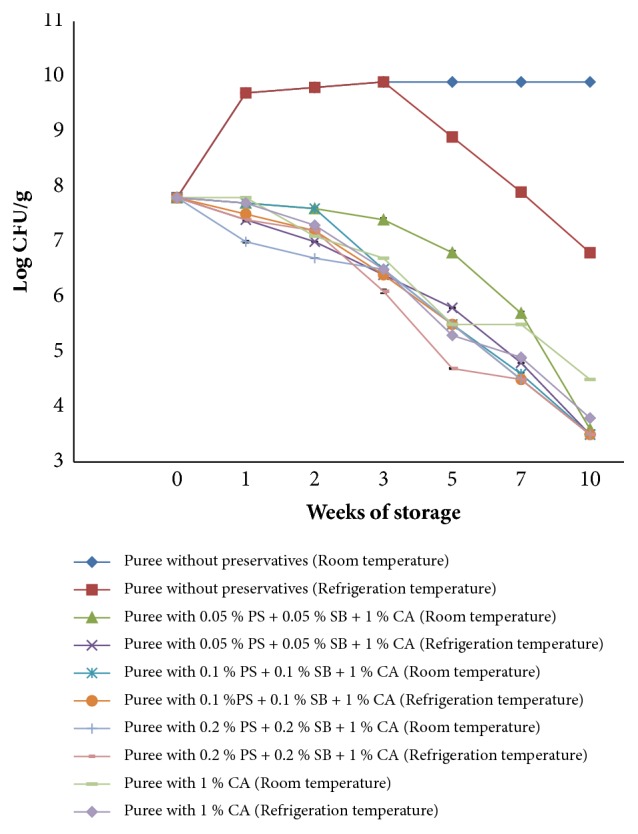
Growth of* E. coli* in OFSP puree with preservatives during storage at ambient temperatures (15-25°C) and refrigeration temperature (4°C).** PS**: potassium sorbate,** SB**: sodium benzoate, and** CA**: citric acid.

Figure 3 shows the growth of* E. coli *in OFSP puree treated with a combination of selected chemical preservatives and that without preservatives and stored at ambient (15-25°C) and refrigeration temperatures (4°C) for 10 weeks.

In nonsupplemented puree,* E. coli* counts increased significantly after inoculation by 2 logs with subsequent increase in storage from 7 log cfu/g to 9 log cfu/g. All combinations of potassium sorbate, sodium benzoate, and 1 % citric acid led to a significant (p<0.05) 4-log reduction in* E. coli* counts in puree kept at ambient temperature from 7 log cfu/g to 3 log cfu/g while 1 % citric acid gave a 3-log reduction from 7 log cfu/g to 4 log cfu/g.

Similarly, preservative treatment with all combinations of potassium sorbate, sodium benzoate, and citric acid led to a significant (p<0.05) reduction of* E. coli* by 4 log cycles (from 7 log cfu/g to 3 log cfu/g) in OFSP puree stored at refrigeration temperature. 1 % citric acid also resulted in a 4-log reduction in the numbers of* E. coli*.* E. coli *counts in nonsupplemented puree increased significantly by 2 logs immediately after inoculation but declined after 3 weeks storage from 7 log cfu/g to 6 log cfu/g. There was no significant difference (p>0.05) in* E. coli* populations in OFSP puree with different combinations of sodium benzoate, potassium sorbate, and citric acid in the two storage conditions.

### 3.3. Growth and Survival of* Staphylococcus aureus* in Stored OFSP Puree

**Figure 4:**
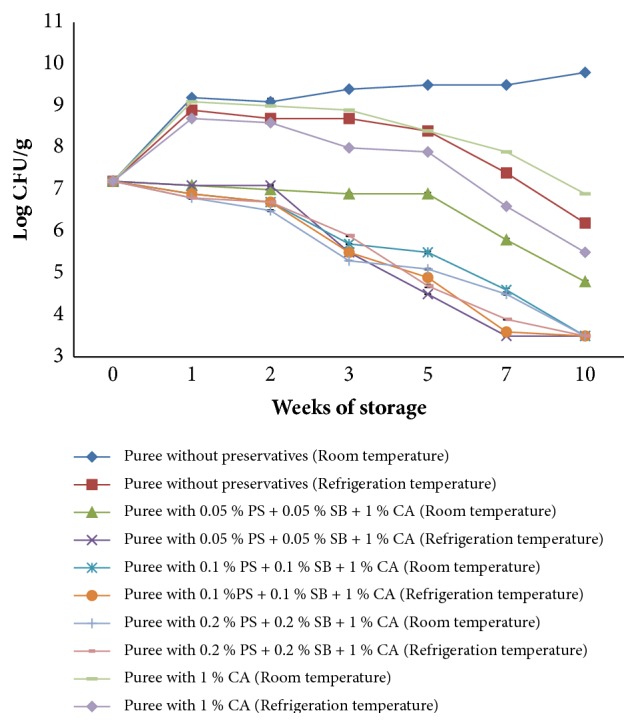
Growth of* S. aureus* in OFSP puree with preservatives during storage at ambient temperatures (15-25°C) and refrigeration temperature (4°C).** PS**: potassium sorbate,** SB**: sodium benzoate, and** CA**: citric acid.

Figure 4 shows the growth of* S. aureus* in OFSP puree treated with a combination of selected chemical preservatives and that without preservatives and stored at ambient (15-25°C) and refrigeration temperatures (4°C) for a period of 10 weeks.

Combined use of sodium benzoate and potassium sorbate at different concentrations with 1 % citric acid led to a 4-log reduction in* S. aureus* counts in OFSP puree kept at ambient and refrigeration temperatures from 7 log⁡cfu/g to 3 log⁡cfu/g.* S. aureus* counts in nonsupplemented puree kept at ambient temperature recorded a 2-log increase immediately after inoculation with subsequent increase in counts during storage from 7 log cfu/g to 9 log cfu/g while that at refrigeration recorded 2-log increase after inoculation with subsequent decline in counts during storage from 7 log cfu/g to 6 log cfu/g.

Treatment with 1 % citric acid recorded a reduction in* S. aureus* population by 1 log cycle at ambient temperature from 7 log cfu/g to 6 log cfu/g and by 2 log cycles at refrigeration temperature from 7 log cfu/g to 5 log cfu/g. Treatment of OFSP puree with potassium sorbate and sodium benzoate at different concentrations together with 1 % citric acid had a slightly greater effect on* S. aureus* growth compared to 1 % citric acid when used alone.

### 3.4. Total Viable Count (TVC) in Stored OFSP Puree

**Figure 5:**
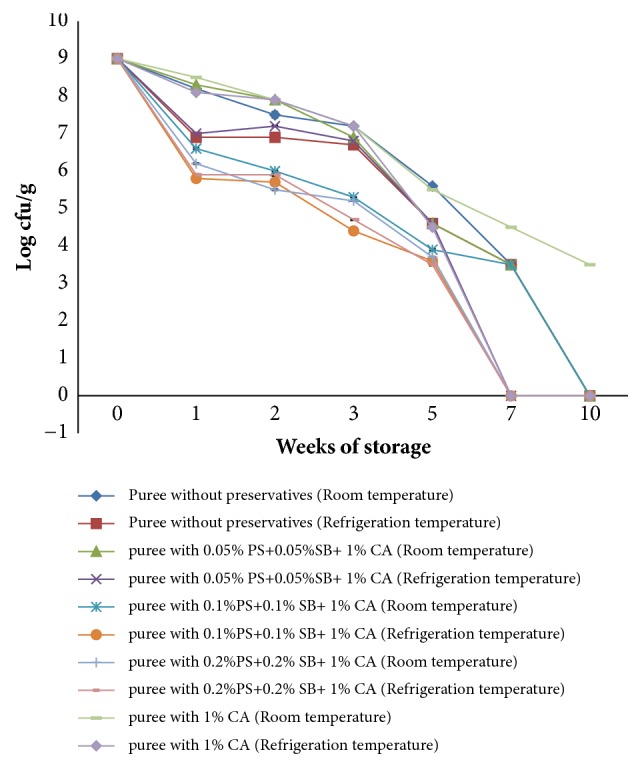
Total aerobic microorganisms in OFSP puree with preservatives during storage at ambient temperatures (15-25°C) and refrigeration temperature (4°C).** PS**: potassium sorbate,** SB**: sodium benzoate, and** CA**: citric acid.

Figure 5 shows the growth of aerobic microorganisms in OFSP puree with and without preservatives and stored at ambient (15-25°C) and refrigeration temperatures (4°C) for 10 weeks.

OFSP puree was found to contain high levels of bacteria (9.0 log cfu/g) immediately after preparation. The counts declined significantly (p<0.05) from 9 log cfu/g to nondetectable levels at the end of the storage period in OFSP puree treated with different combinations of potassium sorbate and sodium benzoate together with 1 % citric acid both at ambient and refrigeration conditions. However, there was a decline in aerobic microorganisms from 9 log cfu/g to 3 log cfu/g in puree with 1 % citric acid at ambient temperature at week 10 of puree storage.

### 3.5. Levels of Yeasts and Molds in OFSP Puree during Storage

**Figure 6:**
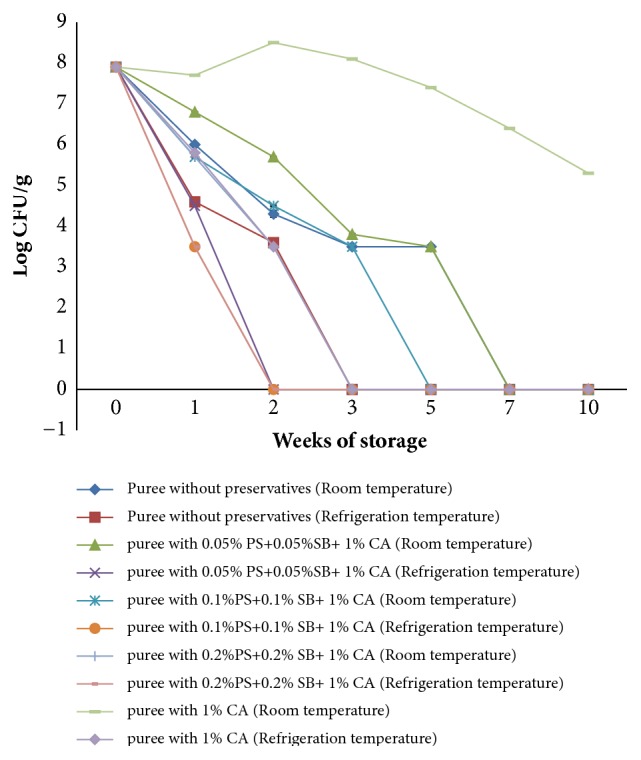
Microbial counts of yeast and molds in OFSP puree with and without preservatives during storage at ambient temperatures (15-25°C) and refrigeration temperature (4°C).** PS**: potassium sorbate,** SB**: sodium benzoate, and** CA**: citric acid.

Figure 6 shows the growth of yeast and molds in OFSP puree with and without preservatives and stored at ambient (15-25°C) and refrigeration temperatures (4°C) for 10 weeks.

The level of yeast and molds counts in OFSP puree after preparation was 7.92 log cfu/g. The counts declined significantly (p<0.05) with storage from 7.92 log cfu/g to nondetectable levels at week 7 of puree storage in all the treatments kept at ambient temperature except for the puree with 1 % citric acid in which yeast and mold counts declined from 7.92 log cfu/g to 5 log cfu/g at week 10 of puree storage. For the puree kept at refrigeration temperature, yeast and mold counts declined significantly (p<0.05) from 7.92 log cfu/g to nondetectable levels at week 3 of puree storage. Pack distention and alcoholic odors were noted in OFSP puree treated with citric acid only after one week of storage.

## 4. Discussion

### 4.1. Effect of PH on Microbial Growth in Orange Fleshed Sweet Potato (OFSP) Puree

Effectiveness of preservative is dependent on pH of the product [26] and pH is also one of the factors that determine the growth and survival of microorganisms during processing and storage [27]. The interest of food processors is to determine the pH of a food product and maintain that pH at a certain level in order to control microbial growth thus preventing product spoilage [28]. The reduction in pH of puree treated with preservatives was mainly because of addition of citric acid. pH is important to the antimicrobial effect of potassium sorbate and sodium benzoate because their effect is due to the undissociated form of their molecule which is dependent on pH [29]. Preservatives such as sodium benzoate and potassium sorbate have also been shown to have an effect on the pH of a food product and therefore the decrease in pH of puree during storage would also be attributed to the presence of preservatives [27].

### 4.2. Growth and Survival of* Escherichia coli* and* Staphylococcus aureus* in OFSP Puree

Information on the survival potential of pathogenic microorganisms in orange fleshed sweet potato (OFSP) puree is limited and such knowledge would be of significance in OFSP puree storage period by the consumer further contributing to food safety and quality. If postprocessing contamination of OFSP puree by* E. coli* and* S. aureus *would occur, the data presented indicate that these pathogens would grow extensively in the puree under ambient temperature assuming a 10-week storage period. The increase in* E. coli* and* S. aureus* counts in nonsupplemented puree (control) can be attributed to nutrient availability and favorable environment for their growth [30] such as pH and water activity.

The decline in* E. coli* and* S. aureus* populations in stored OFSP puree with preservatives can be attributed to various factors. Sodium benzoate and potassium sorbate activity is largely dependent on the pH of a food product. The optimum inhibitory activity of these preservatives takes place at low pH which favors the undissociated form of the molecule that freely moves across the plasma membrane into the cytoplasm [11]. The low pH of the puree was achieved through the addition of citric acid in combination with the preservatives. Due to the neutral pH of the cytoplasm, the acid dissociates into anions and protons. These molecules are not able to diffuse back across the cell membrane and hence accumulate in the cytoplasm. Acidification of the cytoplasm and the energy depletion lead to physiological malfunction finally inhibiting microbial growth [31, 32]. In addition to the preservatives, the storage conditions and vacuum packaging of the puree also contributed towards inactivation of* E. coli *and* S. aureus* in the puree. The results are similar to those reported by Chikthimmah [33], who demonstrated that the use of chemical preservatives was critical for a significant reduction in* E. coli* counts in apple cider stored under ambient and refrigeration conditions.

The antimicrobial activity of citric acid was due to reduction of pH of OFSP puree below the optimal range of pH values for* E. coli* and* S. aureus* growth which is 6-7 or due to the disruption of the pathogens' membrane permeability thus preventing entry of essential nutrients for its growth. Other researchers have demonstrated the effectiveness of citric acid in inhibiting the growth of* S. aureus*. For instance, Seo [34] found that 2 % citric acid was effective in reducing counts of* S. aureus* in chicken meat in 5 days. Abu-ghazaleh [35] showed that 0.03 % citric acid significantly inhibited* S. aureus* growth in growth medium after 24 hours of incubation. Similarly, the use of 2 % citric acid alone on chicken meat led to the decline in* E. coli* counts by 4 log cycles in 12 hours [34].

There were no significant differences (p>0.05) in* E. coli* and* S. aureus* populations in OFSP puree with different combinations of sodium benzoate, potassium sorbate, and citric acid in the two storage conditions. This suggests that even the lowest concentration of preservatives used in combinations was effective in inhibiting the growth of* E. coli* and* S. aureus* in OFSP puree during the storage period. There was a slightly better inhibition of* E. coli *and* S. aureus *in treatments with different combinations of potassium sorbate, sodium benzoate, and citric acid as compared to citric acid alone at ambient and refrigeration temperatures. This suggests that combination of a number of hurdles (preservative factors) gives higher or multiple inhibitory effects against microorganisms compared to a single hurdle. According to Lotte Dock [29], the effect of combined treatments with preservatives in apple cider was significantly greater than that of a single preservative used alone. For instance, antimicrobial activity against* E. coli* was enhanced through the combined use of 0.1 % potassium sorbate and 0.1 % sodium benzoate at 8°C with survival time being reduced by 50 % compared with the one with 0.1 % sodium benzoate alone [29].

Temperature is also known to be one of the significant factors affecting microbial growth in food products [36]. The enzyme activity of microorganisms is optimum at a certain temperature range beyond which the enzyme undergoes denaturation; thus microbial growth was inhibited. As expected, growth of* S. aureus* and* E. coli* was more rapid at 25°C compared to 4°C. This is because, at low temperatures, the fluidity of the cytoplasmic membrane of microorganisms is reduced, thus interfering with transport mechanisms [37]. Therefore, microbial growth rate increases with increasing temperature until the maximum temperature for growth is attained [38].* E. coli* is able to grow at a temperature range of 4-45°C with an optimum of 37°C but can survive refrigeration and freezing temperatures. A study carried out on the effect of temperature on the growth of* E. coli* revealed that it can grow and survive well on a range of temperatures but can grow well at 37°C compared to other temperatures [39].* S. aureus* on the other hand grows on a temperature range of 7-48°C with an optimum of 37°C [40].

### 4.3. Growth of Aerobic Microorganisms, Yeast, and Molds in OFSP Puree

The high levels of aerobic microorganisms in OFSP puree before treatment with preservatives would be attributed to poor handling during preparation. The reduction in levels of aerobic counts in OFSP puree samples treated with different combinations of potassium sorbate, sodium benzoate, and citric acid indicates the benefits of the combination of antimicrobial chemicals having multiple effects against bacterial growth in OFSP puree. Vacuum packaging of the puree eliminates oxygen which is essential for the growth of aerobic microorganisms thus eliminating them even in the nonsupplemented puree. Other researchers showed the effect of preservatives on the growth of aerobic microorganisms. For instance, Ogiehor & Ikenebomeh [41] recorded a decline in aerobic microorganisms in Garri product treated with 0.2 % sodium benzoate stored for 6 months at 30°C. Results obtained by Momoh [42] showed that 0.1 % sodium benzoate along with refrigeration was able to inhibit aerobic microorganism multiplication of up to 13 days of storage while at room temperature the inhibition lasted for only 4 days.

The complete inhibition of yeast and molds in OFSP puree with and without preservatives would be attributed to the vacuum packaging of the puree that eliminates oxygen and therefore prevents the growth of molds and oxidative yeasts since they do not grow in the absence of oxygen [43]. However, yeast and molds counts were still detected in puree with 1 % citric acid and kept at ambient temperature even at week 10 of puree storage. Pack distention and alcoholic odors were noted in OFSP puree treated with citric acid only after one week of storage. This could be attributed to the growth of fermentative yeasts and/or lactic acid bacteria in the puree metabolizing simple sugars into ethanol and carbon dioxide. According to Rawat [44], yeasts can grow at very low pH values. Other researchers have demonstrated the effect of preservatives on fungal growth in food products. For instance, Omojowo [45] reported that 3-5 % potassium sorbate led to a decline in levels of yeast and molds in smoked fish stored for 8 weeks. A study by Guynot [46] demonstrated that potassium sorbate at concentrations of 0.15-0.30 % was effective in preventing fungal growth.

## 5. Conclusion

The study sought to investigate the effect of different combinations of preservatives on microbial growth in OFSP puree at different storage conditions and time. Different combinations of sodium benzoate, potassium sorbate, and citric acid improved microbial keeping quality and inhibited the growth of* Escherichia coli* and* Staphylococcus aureus* in OFSP puree as indicated by the microbial challenge test. Use of citric acid alone was less effective in controlling the growth of these pathogens.

## Figures and Tables

**Table 1:** Bacteria counts in OFSP puree (log cfu/g).

	**Raw OFSP puree (log cfu/g)**	**OFSP puree after microwaving (Log cfu/g)**
***S. aureus***	6.9 ± 0.04	nd*∗*
***E. coli***	5.7 ± 0.05	nd*∗*

Each value is mean ± standard deviation for triplicate experiments. nd*∗*: not detected.

**Table 2:** Treatments of OFSP puree with combination of selected chemical preservatives.

**Sample ID**	**Treatments**
A	Puree without preservatives
B	Puree with 0.05 % sodium benzoate + 0.05 % potassium sorbate + 1 % citric acid
C	Puree with 0.1 % sodium benzoate + 0.1 % potassium sorbate + 1 % citric acid
D	Puree with 0.2 % sodium benzoate + 0.2 % potassium sorbate + 1 % citric acid
E	Puree with 1 % citric acid
